# Practice of Pain Management by Indian Healthcare Practitioners: Results of a Paper Based Questionnaire Survey

**DOI:** 10.1155/2015/891092

**Published:** 2015-08-23

**Authors:** Gauri Billa, Mukesh Gabhane, Swati Biswas

**Affiliations:** Abbott Healthcare Pvt. Ltd., 1st Floor, D Mart Building, Mulund-Goregaon Link Road, Mumbai 400080, India

## Abstract

*Objective*. Understanding factors while selecting an analgesic and its usage pattern by Indian healthcare practitioners (HCPs). *Methods*. Questionnaire-based survey was conducted among six healthcare specialties. *Results*. Total 448 HCPs participated. Patient's age (72.8%, 74.4%, 87.5%, and 78.9%) and duration of therapy (70.8%, 66.2%, 69.6%, and 73.6%) were main attributes for selecting an opioid according to general practitioners (GPs), dentists, consulting physicians (CPs), and surgeons, respectively. Patient's age was important factor while selecting NSAID according to 77.60%, 66.91%, and 84.20% of GPs, dentists, surgeons, respectively. For mild pain, paracetamol was the choice according to 77%, 78.57% and 74% of GPs, CPs, and surgeons, respectively. For moderate pain, 77%, 87.50%, 68%, and 80.30% of GPs, CPs, surgeons and orthopedicians, respectively, preferred the use of paracetamol + tramadol combination. For moderate pain, NSAID + paracetamol and paracetamol+diclofenac were used by 68.94% and 47.73% of orthopedicians, respectively. Lack of pain clinic (38.8%) in city was commonly cited reason for not referring patients to pain clinics. *Conclusion*. Patient's age, duration of therapy, comorbid conditions, frequency of dosing, and severity of pain are important parameters while selecting analgesics. Paracetamol and its combinations are commonly used for mild and moderate pain, respectively. Pain clinics currently have limited presence in India.

## 1. Introduction

Regardless of age, sex, and region, pain is a significant health issue worldwide [1]. Everybody suffers from some type of pain during life time. Understanding pain as a disease [1], better diagnosis, and treatment may help to reduce overall health burden associated with pain. Currently, numerous nonpharmacological and pharmacological treatment options are available for the management of pain. The major pharmacological options include paracetamol, nonsteroidal anti-inflammatory drugs (NSAIDs), and opioids. In many cases, combination of analgesics is required for effective pain relief. Unfortunately, no single agent is an ideal choice for all types of patients or no one agent is suitable for all types of pain [2]; hence physician has to choose the best suitable agent from the available options based on different factors including patient dynamics, medicine related factors, and disease related aspects. Patient satisfaction also plays an important role in ensuring compliance with the treatment, especially during long term treatment. Understanding physician's perspective while selecting an analgesic and significant barriers in effective pain management through a systematic approach and addressing them with appropriate measures could help for better outcomes.

## 2. Objective

The objective of this study was to understand the attributes for selection of analgesics and usage pattern of analgesics for different pain conditions by Indian healthcare practitioners (HCPs).

## 3. Material and Methods

A cross-sectional, paper based survey was conducted among HCPs across India. Healthcare professionals practicing in India were approached in their working set-ups for participation in the survey. A predefined questionnaire was administered to HPCs of six different disciples: general practitioners (GPs), consulting physicians (CPs), dentists, neurologists, orthopedicians, and general surgeons. The questions were divided into the following three sections:

- Factors considered while selecting opioids and NSAIDs.
- Choices of different analgesics.
- Limitations for referral of selected patients to the pain clinic.

Suggestions from HCPs for better pain management were also recorded. Completed survey forms were collected by the representatives of the company.

## 4. Statistical Analysis

The number and percentage of HCPs responses for each question were calculated. Missing data was not considered for calculating percentages. SPSS version 19.00 was used for the statistical analyses.

## 5. Results

A total of 448 healthcare practitioners across India were enrolled for participation in the study. Specialty-wise distribution of survey participants is given in Table 1.

As neurologists constituted very small number of total survey population, the results from this discipline are not reported in this paper.

### 5.1. Factors Considered While Selecting an Opioid Agent

**Figure 1:**
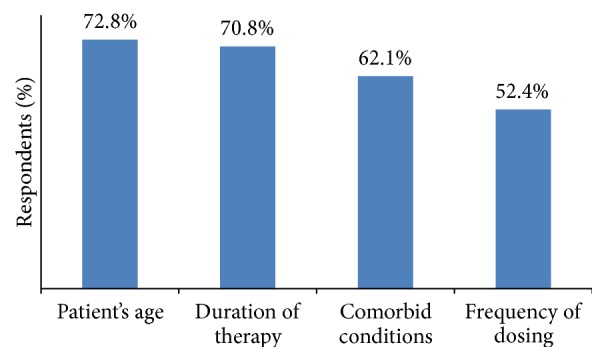
Factors considered by GPs while selecting opioids.

Patient's age, duration of therapy, comorbid conditions, and frequency of dosing were the main attributes for use of opioid according to 72.8%, 70.8%, 62.1%, and 52.4% of GPs, respectively (Figure 1).

**Figure 2:**
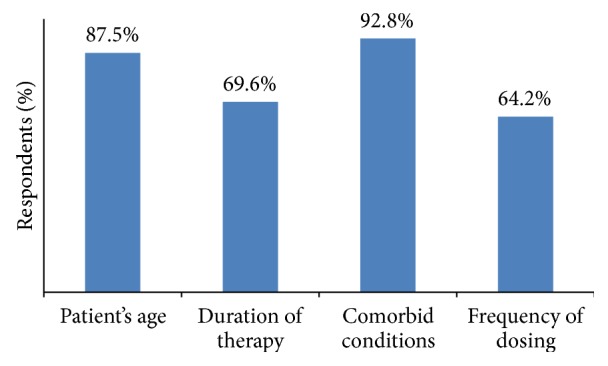
Factors considered by CPs while selecting opioids.

According to 92.8% of CPs, comorbid condition was the most important factor for selecting an opioid for analgesia. Patients age, duration of therapy, and frequency of dosing were considered as important parameters by 87.5%, 69.6%, and 64.2% of CPs, respectively (Figure 2).

**Figure 3:**
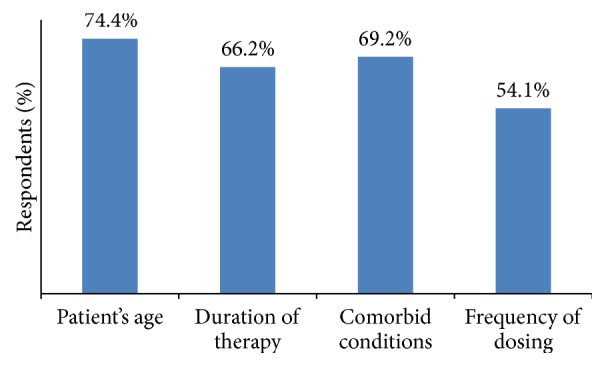
Factors considered by dentist while selecting opioids.

Patient's age, comorbid conditions, duration of therapy, and frequency of dosing were considered as main factors while selecting an opioid by 74.44, 69.2%, 66.2%, and 54.1% of dentists, respectively (Figure 3).

**Figure 4:**
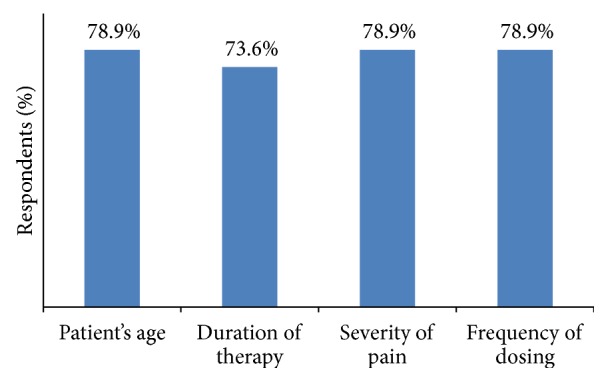
Factors considered by general surgeons while selecting opioids.

Patients age, duration of therapy, severity of pain, and frequency of dosing were the important attributes for selecting opioid analgesic according to 78.9%, 73.6%, 78.9%, and 78.9% of general surgeons, respectively (Figure 4).

### 5.2. Factors Considered While Selecting NSAIDs

**Figure 5:**
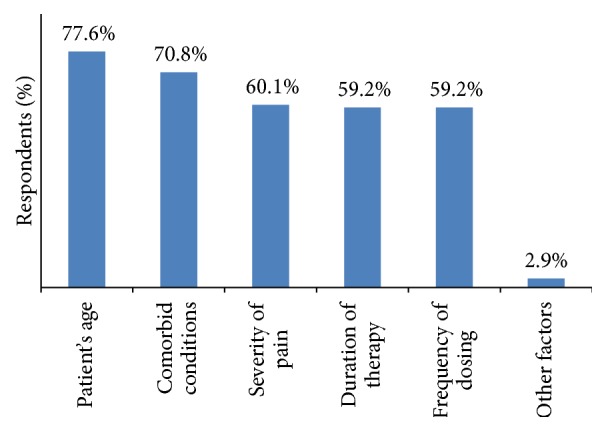
Factors considered by GPs while selecting NSAIDs.

Patient's age (77.6%), comorbid conditions (70.8%), severity of pain (60.1%), duration of therapy, frequency of dosing (59.2% each), and other factors such as cost, gender, and social issues (2.9%) were the major attributes reported by GPs while selecting NSAIDs (Figure 5).

**Figure 6:**
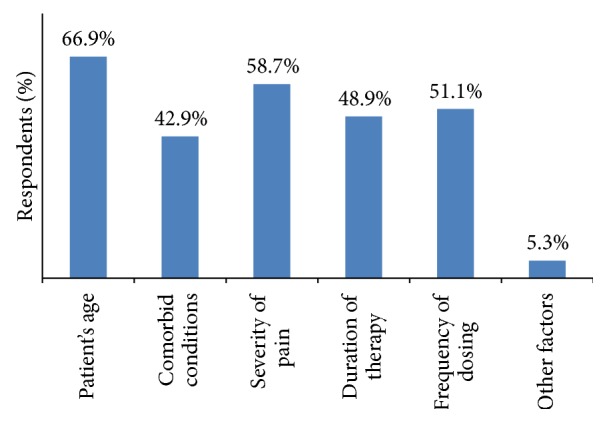
Factors considered by dentists while selecting NSAIDs.

Patient's age (66.9%), comorbid conditions (42.9%), severity of pain (58.7%), duration of therapy (48.9%), frequency of dosing (51.1%), and other factors such as cost, gender, and social issues (5.26%) were the major attributes reported by dentists for selection of NSAIDs (Figure 6).

**Figure 7:**
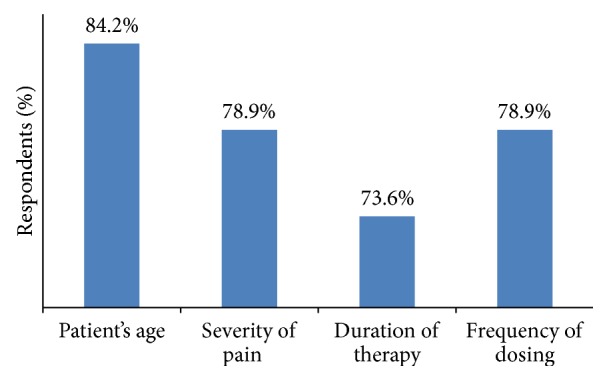
Attributes for the use of NSAIDs according to general surgeons.

Patient's age (84.2%), severity of pain (78.9%), duration of therapy (73.6%), and frequency of dosing (78.9%) were the major attributes shared by general surgeons during selection of NSAIDs (Figure 7).

For mild pain, paracetamol was the choice of analgesic by 77% ofGPs while for moderate pain 77% of GPs reported use of paracetamol plus tramadol combination. For the treatment of severe pain, nonspecific NSAIDs were choice of 64% of GPs. The details of other analgesics preferred by GPs for the management of mild, moderate, and severe pain are enlisted in Table 2.

Paracetamol was the choice of analgesic of 78.57% of CPs in the management of mild pain. The combination of paracetamol plus tramadol was preferred by 87.50% of CPs for the treatment of moderate pain. Nonspecific NSAIDs were preferred by 57.14% of CPs in the management of severe pain.  Table 3 gives list of different analgesics used by CPs for the management of mild, moderate, and severe pain.

Nonspecific NSAIDs were preferred by 88.64% of orthopedicians for the management of mild pain. For the moderate pain, the combination of tramadol plus paracetamol was preferred by 80.30% of orthopedicians while NSAID plus paracetamol and paracetamol plus diclofenac were favored by 68.94% and 47.73% of orthopedicians, respectively.

The list of different analgesics preferred by orthopedicians and general surgeons for the management of mild, moderate, and severe pain is given in Tables 4 and 5.

Lack of pain clinic in the city, cost of treatment at pain clinic, and long travelling distance were commonly reported reasons for not referring the patients for pain clinics (Table 6).

Multimodal analgesia, patient controlled analgesia, referral to pain specialists, use of special techniques, and patient education and counseling were suggested by healthcare professions for better pain management (Table 7).

## 6. Discussion

Pain is one of the most common health problems for which patients seek consultation from the HCP, often after using over-the-counter medications. There is confusion about efficacy and safety of common analgesics [3] which contributes to dilemma while selecting one agent over the other. To understand different parameters considered by HPCs in real-life clinical practice while selecting an analgesic, we conducted a nationwide survey among six healthcare disciplines (general physicians, consulting physicians, orthopedic surgeons, general surgeons, dental clinicians, and neurologists) in India.

Patient's age was the common factor considered by all surveyed healthcare disciplines while selecting an analgesic for the management of pain. The other important criteria for analgesic selection included duration of therapy and frequency of dosing which have potential to improve the patient compliance [4]. While selecting NSAIDs, severity of pain and duration of therapy were considered equally important by most of the healthcare practitioners.

The patients seeking consultation could have another underlying systemic disease; hence careful history of comorbid conditions is important while prescribing an analgesic to avoid complications. For example, NSAIDs can cause GI, haematological, or renal adverse events [2]. Comorbid conditions were considered as an important attribute while selecting an opioid analgesic by dentists, GPs, and CPs, while GPs and dentists also consider comorbid conditions as an important attribute while selecting NSAIDs. Paracetamol is an important component of pain management [2]. It is a good alternative to NSAIDs because of less adverse events [5]. Usually it does cause adverse events except with overdosage [6]. According to the results of our study, paracetamol is the preferred analgesic for the management of mild pain by all healthcare disciplines surveyed except orthopedicians. Most orthopedicians mainly use nonspecific NSAIDs for the management of mild pain. GPs, CPs, and orthopedicians also commonly use muscle relaxants for the management of mild pain. The reason of common use of skeletal muscle relaxants by these HCPs could be related to the higher number of patients with musculoskeletal spasm visiting them compared to others.

Opioids are commonly used for treating moderate to severe pain [7]; however strong opioids are not commonly required for the management of musculoskeletal pain [8] or postoperative pain such as ambulatory hand surgery [9]. Tramadol, a synthetic, centrally acting analgesic with weak opioid agonist action, does not cause clinically significant adverse effects on respiratory or cardiovascular system at the recommended dose [10]. Analgesic combination with complementary mechanisms (e.g., tramadol plus paracetamol) is often used for better efficacy and safety compared to individual agents [9]. We observed very common use of combination therapy especially paracetamol based combination with either tramadol or NSAID in the management of moderate pain. Paracetamol and NSAIDs or tramadol act by different mechanisms and hence provide complimentary mechanisms of action to each other. Tramadol is preferred over other opioids in combination treatment due to its unique mechanism of action and better safety profile [2]. NSAIDs and selective cyclooxygenase-2 inhibitors can reduce opioid use [11]. Recently, in Indian patients, combination of diclofenac, one of the routinely used NSAIDs with tramadol, has been shown effective and well tolerated in the management of pain because of acute musculoskeletal conditions, acute flare of osteoarthritis or rheumatoid arthritis, and postoperative pain [12].

Dental clinicians commonly select analgesics based on the pharmacodynamics and safety profile of the medicinal product [13] and use them for the management of intra-/postoperative pain and acute/chronic pain [14]. According to our study, patients age, comorbid conditions, duration of therapy, and frequency of dosing were considered to be important factors while selecting an opioid by dentists.

According to a pan European survey among primary care physicians, use of pain assessment tools, improving confidence for using opioids, and having guidelines for the management of chronic nonmalignant pain are the areas for improvement [15]. About 8% of dental clinicians in this survey also expressed the need for national guidelines on the pain management.

Though pain clinic is an important referral center for nonresponding patients, limited access and cost are major barriers for referring patients to these clinics, according to HCPs surveyed. Patient education and counseling can influence the outcome of pain management strategies and hence should be routinely practiced according to large number of GPs, CPs, and dentists.

The study holds limitations of an observational and cross-sectional design. Moreover, the survey forms were provided and collected by the company representative; hence reporting bias cannot be ruled out. Nonrandom sampling may not represent the entire specialty; hence the results of this survey should be carefully extrapolated.

## 7. Conclusion

According to the findings of the present survey, patient's age, duration of therapy, comorbid conditions, frequency of dosing, and severity of pain are the main factors for the selection of analgesic. Paracetamol and paracetamol based combination are preferred for mild and moderate pain, respectively.

## Figures and Tables

**Table 1:** Distribution of survey participants.

Specialty	*N* (%)
General practitioners	103 (23.0%)
Consulting physicians	56 (12.5%)
Dentists	133 (29.7%)
Orthopedicians	132 (29.5%)
General surgeons	19 (4.2%)
Neurologists	5 (1.1%)
**Total**	**448 (100%)**

**Table 2:** Choice of analgesics for pain management by GPs.

Mild pain		Moderate pain		Severe pain	
Paracetamol	77%	Tramadol + paracetamol	77%	Nonspecific NSAID	64%
Paracetamol + NSAID with supportive therapy	75%	NSAID + paracetamol	61%	Strong opioid injection followed by oral opioid	53%
Topical NSAID	70%	NSAID + muscle relaxants	58%	Cox 2 selective NSAID	43%
Skeletal muscle relaxant	70%	Paracetamol + Diclofenac	56%	Strong opioid injection followed by oral NSAID	46%
NSAID + paracetamol	68%	Topical NSAID with oral NSAID	54%	Strong opioid injection followed by oral NSAID + paracetamol	43%
Pain modulators	68%	Cox 2 selective NSAID	49%	Smooth muscle relaxants	43%
Cox 2 selective NSAID	59%	Nonspecific NSAID	49%	Strong opioid injection followed by oral mild opioid + paracetamol	42%
Smooth muscle relaxants	59%	Paracetamol + muscle relaxants	47%	Mild opioid + diclofenac	41%
Nonspecific NSAIDs	58%	Mild opioid + paracetamol	40%		
		Mild opioid + diclofenac	40%		

**Table 3:** Choice of analgesics for pain management by CPs.

Mild pain		Moderate pain		Severe pain	
Paracetamol	78.57%	Nonspecific NSAIDs	37.50%	Nonspecific NSAIDs	57.14%
Paracetamol/NSAIDs with supportive therapy	66.07%	Cox2 selective NSAIDs	44.64%	Cox2 selective NSAIDs	42.86%
Nonspecific NSAIDs	53.57%	NSAIDs + paracetamol	71.43%	Strong opioids inj. followed by oral opioid	50%
NSAIDs − paracetamol combination	53.57%	Topical NSAID with oral NSAIDs	46.43%	Strong opioids inj. followed by oral NSAIDs	53.57%
Topical NSAID	58.93%	Tramadol + paracetamol	87.50%	Strong opioids inj. followed by NSAIDs − paracetamol	39.29%
Cox-2 selective NSAIDs	58.93%	Paracetamol + diclofenac	35.71%	Inj. followed by oral mild opioid paracetamol combination	48.21%
Skeletal muscle relaxant	75%	Mild opioid + paracetamol	35.71%	Mild opioid + diclofenac	32.14%
Smooth muscle relaxant	41.07%	Mild opioid + diclofenac	25%	NSAID − muscle relaxant	30.36%
Pain modulators	76.79%	NSAID − muscle relaxant	48.21%	** **	** **
** **	** **	Paracetamol + muscle relaxant	28.57%	** **	** **

**Table 4:** Choice of analgesics for pain management by orthopedicians.

Mild pain		Moderate pain		Severe pain	
Paracetamol	64.39%	Nonspecific NSAIDs	46.97%	Cox-2 selective NSAIDs	61.36%
Nonspecific NSAIDs	88.64%	NSAIDs + paracetamol	68.94%	Strong opioids injectable followed by oral NSAID	46.21%
Topical NSAID	58.33%	Tramadol + paracetamol	80.30%	Strong opioids injectable followed by NSAID + paracetamol	55.30%
Muscle relaxant	71.21%	Paracetamol + diclofenac	47.73%	Strong opioids injectable followed by oral mild opioid + paracetamol	50.76%
Pain modulators	63.64%	Pain modulators	49.24%	Strong opioids injectable followed by oral mild opioid + diclofenac	46.21%
		Topical NSAID	46.97%	Pain modulators	34.09%
		Muscle relaxant	54.55%	Intra-articular steroids	56.06%

**Table 5:** Choice of analgesics for pain management by general surgeons.

Mild pain		Moderate pain		Severe pain	
Paracetamol	74%	Tramadol + paracetamol	68%	Nonselective NSAIDs	84%
Nonspecific NSAIDs	68%	Pain modulators	63%	Cox 2 selective NSAID	53%
COX 2 selective NSAIDs	53%	NSAID + paracetamol	58%	Strong opioid injection followed by oral mild opioids + paracetamol	53%
Antispasmodic agents	53%	Paracetamol + diclofenac	58%	Strong opioid injection followed by oral NSAIDs + paracetamol	47%
Pain modulators	53%	Antispasmodic agents	58%	Intra-articular steroids	42%
		Nonspecific NSAIDs	53%	Strong opioid injection followed by oral mild opioids + diclofenac	42%
		Cox 2 selective NSAIDs	47%	Strong opioid injection followed by oral NSAIDs	42%
				Pain modulators	32%

**Table 6:** Common limitations for referral to pain clinics.

Reason	Percentage of healthcare practitioners
Lack of pain clinic in the city	38.8%
Cost of treatment	36.6%
Distance from home or work place	26.1%

**Table 7:** Suggestions by healthcare professionals for better pain management.

GPs	CPs	General surgeons	Orthopedicians	Dentists
(i) Multimodal analgesia (84%) (ii) Patient controlled analgesia (51%) (iii) Referral to pain specialists (50%) (iv) Use of special techniques (30%) (v) Patient education (54%) (vi) Counseling (30%)	Patient education (29%)	(i) Multimodal analgesia (63.84%) (ii) Patient controlled analgesia (36.8%) (iii) Referral to pain specialists (31.57%) (iv) Use of special techniques (21%)	(i) Multimodal analgesia (83%) (ii) Patient controlled analgesia (32%) (iii) Referral to pain specialists (21%) (iv) Use of special techniques (51%)	(i) Patient education (45.11%) (ii) Patient counseling (20.30%) (iii) Treatment guidelines (8.17%)
